# Protective Effect of *Opuntia dillenii* Haw Fruit against Lead Acetate-Induced Hepatotoxicity: *In Vitro* and *In Vivo* Studies

**DOI:** 10.1155/2021/6698345

**Published:** 2021-04-29

**Authors:** Reza Shirazinia, Ali Akbar Golabchifar, Vafa Baradaran Rahimi, Abbas Jamshidian, Alireza Samzadeh-Kermani, Parisa Hasanein, Mohammadreza Hajinezhad, Vahid Reza Askari

**Affiliations:** ^1^Department of Comparative Biosciences, Faculty of Veterinary Medicine, University of Tehran, Tehran, Iran; ^2^Applied Biomedical Research Center, Mashhad University of Medical Sciences, Mashhad, Iran; ^3^Pathobiology Department, Veterinary Faculty, University of Zabol, Zabol, Iran; ^4^Chemistry Department, Basic Science Faculty, University of Zabol, Zabol, Iran; ^5^Department of Biology, School of Basic Sciences, University of Zabol, Zabol, Iran; ^6^Department of Basic Science, School of Veterinary Medicine, University of Zabol, Zabol, Iran; ^7^Department of Pharmaceutical Sciences in Persian Medicine, School of Persian and Complementary Medicine, Mashhad University of Medical Sciences, Mashhad, Iran; ^8^Department of Persian Medicine, School of Persian and Complementary Medicine, Mashhad University of Medical Sciences, Mashhad, Iran

## Abstract

Lead is one of the most common environmental contaminants in the Earth's crust, which induces a wide range of humans biochemical changes. Previous studies showed that *Opuntia dillenii* (*OD*) fruit possesses several antioxidant and anti-inflammatory properties. The present study evaluates *OD* fruit hydroalcoholic extract (OHAE) hepatoprotective effects against lead acetate- (Pb-) induced toxicity in both animal and cellular models. Male rats were grouped as follows: control, Pb (25 mg/kg/d i.p.), and groups 3 and 4 received *OHAE* at 100 and 200 mg/kg/d + Pb (25 mg/kg/d i.p.), for ten days of the experiment. Thereafter, we evaluated the levels of alkaline phosphatase (ALP), alanine aminotransferase (ALT), and aspartate aminotransferase (AST), catalase (CAT) activity and malondialdehyde (MDA) in serum, and liver histopathology. Additionally, the cell study was also done using the HepG2 cell line for measuring the direct effects of the extract on cell viability, oxidative stress MDA, and glutathione (GSH) and inflammation tumor necrosis factor-*α* (TNF-*α*) following the Pb-induced cytotoxicity. Pb significantly increased the serum levels of ALT, AST, ALP, and MDA and liver histopathological scores but notably decreased CAT activity compared to the control group (*p* < 0.001 for all cases). *OHAE* (100 and 200 mg/kg) significantly reduced the levels of serum liver enzyme activities and MDA as well as histopathological scores while it significantly increased CAT activity compared to the Pb group (*p* < 0.001–0.05 for all cases). *OHAE* (20, 40, and 80 *μ*g/ml) concentration dependently and significantly reduced the levels of MDA and TNF-*α*, while it increased the levels of GSH and cell viability in comparison to the Pb group (*p* < 0.001–0.05 for all cases). These data suggest that *OHAE* may have hepatoprotective effects against Pb-induced liver toxicity both *in vitro* and *in vivo* by its antioxidant and anti-inflammatory activities.

## 1. Introduction

Lead (Pb) is the most common environmental contaminants in the Earth's crust, which can induce a wide range of toxicity, including biochemical, physiological, and behavioral changes in humans by multiple mechanisms [1]. Indeed, lead pollution and poisoning are common problems in industrial and developing countries [2]. Heavy metals, especially lead, are highly stable in the environment and can be concentrated in the food chains [3]. World health organization (WHO) recently declared that, based on 2016-2017 reports, lead intoxication is responsible for more than one million deaths throughout the world, with the highest burdens in the developing countries. Based on the Institute for Health Metrics and Evaluation (IHME), about 63.2% of idiopathic developmental, intellectual disabilities, 10.3% of hypertensive heart diseases, 5.6% of ischemic heart diseases, and 6.2% of strokes were related to lead exposure [4]. Lead is present in every food grain and can enter the body through drinking contaminated water or breathing polluted air. Even some cosmetics may contain tiny amounts of lead; therefore, lead pollution exists in every aspect of our daily life [5].

The central nervous system, hematopoietic system, liver, and kidney systems are the considered main targets of lead toxicity. Lead enters the body by different routes, through eating (main route) or inhalation, and then, it is circulated via blood and stored in the soft tissues and bone [6, 7]. Even continuous exposure to low levels of lead acetate as 1.0 *μ*g/g can affect the levels of intelligence, behavior, attention, and growth [7, 8]. It has been shown that the liver cannot metabolize lead acetate and also excrete it adequately; therefore, this metal is distributed in the body and gradually accumulated in the various organs, including bone, muscle, brain, liver, kidney, hematopoietic system, central nervous system, and gastrointestinal tract [9]. Contextually, lead accumulation in the body causes several impairments and imbalances in the body, including oxidants and antioxidants, and immunity systems [10, 11]. Lead-induced oxidative stress in blood and other tissues is the major mechanism of lead toxicity [12]. Moreover, lead intoxication causes activation of inflammatory cells that may provoke inflammation and related diseases [13, 14]. Indeed, lead exposure causes a significant increase in the level of blood inflammatory biomarkers, including interleukin 1-*β* (IL-1*β*), interleukin-6 (IL-6), and TNF-*α* [15]. Chronic lead poisoning is not detectable in 29% of patients, but hypertension, gout, chronic renal failure, and hypothyroidism and impotence are considered important diagnosis factors [16–18].

**Figure 1:**
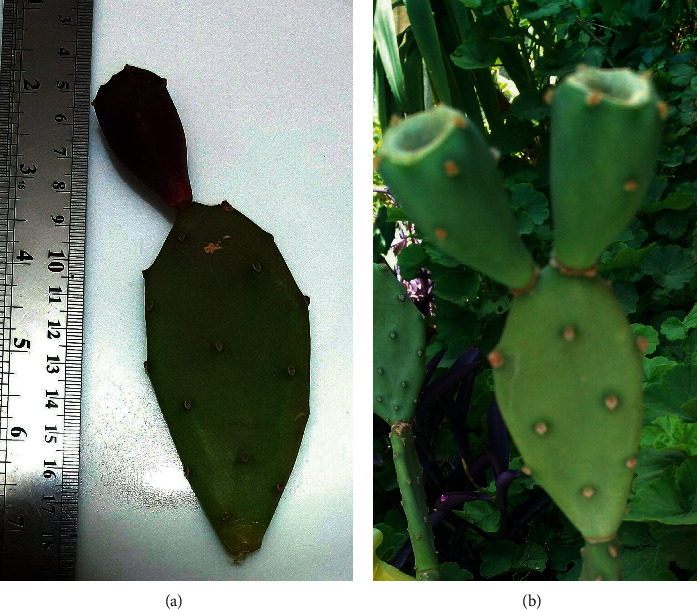
*Opuntia dillenii* plant and fruit. The top-right shows the ripe fruit.

*Opuntia dillenii* (*OD*), a member of Cactaceae (Figure 1), is widely grown in tropical/subtropical areas such as China, India, and Iran [19–21]. People in Asia have adopted *OD* as a medicinal plant. In India, it is used to treat gastrointestinal disease, pimples, and syphilis [22]. Several ingredients, including polyphenols, polysaccharides, flavonoids, dietary fibers, and vitamin C (ascorbic acid), have been isolated from the fruits of *OD* [23]. OD's protective effect and the other plants of *Opuntia* species have been investigated against heavy metal-induced toxicity in previous studies [20, 21]. Moreover, *OD* has shown the potential bioabsorption and removal capacity against heavy metals intoxication [24–26]. Previous studies also have indicated *OD* as a plant with desirable pharmacologic properties including anti-inflammatory and immunomodulatory [27, 28], antioxidant [29], neuroprotective [30], antidiabetic [31], analgesic [28], antitumor [32], hypotensive [33], antimicrobial [34], and hepatoprotective effects [34]. Loro et al. reported that intraperitoneal (i.p.) administration of *OD* ameliorates carrageenan-induced rat paw edema as a model of inflammation in a dose-dependent manner [28]. Moreover, crude polysaccharides obtained from *OD* possessed antioxidant properties evidenced by DHPP assay [29]. Hitherward, there is no report regarding the protective effects of *OD* against heavy metal-induced liver, oxidative, and inflammatory damages. Therefore, we investigated OD fruit extract's possible protective effect on lead-induced hepatotoxicity, oxidative stress, and inflammation in both *in vivo* and *in vitro* models.

## 2. Material and Methods

### 2.1. Drugs and Chemicals

Ethanol was purchased from Sigma (USA). Lead acetate was purchased from Merck (Germany). Aspartate transaminase (AST), alanine aminotransferase (ALT), and alkaline phosphatase (ALP) kits were purchased from Pars Azmoon Company (Tehran, Iran). TBARS assay kit was provided from BioAssay Systems (Hayward, CA 94545, USA). Catalase (CAT), glutathione (GSH), and malondialdehyde (MDA) assay kits were purchased from ZellBio (Germany). HepG2 cell line was from Pasteur Institute (Tehran, Iran). Dulbecco's Modified Eagle Medium (DMEM)/F12 culture media, penicillin, and streptomycin (pen/strep), amphotericin B, fetal bovine serum (FBS), and L-glutamine were purchased from Sigma-Aldrich Chemical Co. (St. Louis, MO, USA). TNF-*α* assay kit was purchased from Bender Med (Germany). All other materials were analytical and cell culture grade was provided from Sigma-Aldrich Chemical Co. (St. Louis, MO, USA).

### 2.2. Plant Material

#### 2.2.1. Preparation of the Extracts

*OD* (Nagphana) fresh fruits were collected from a local market in May 2017 from Zahedan, Sistan, and Baluchestan Province, Iran. The plant was identified by Mrs. Sozani, and the voucher samples were deposited in the School of Pharmacy herbarium, Mashhad University of Medical Sciences, Iran (No. 13161).

Extraction was conducted based on the method described by previous studies with slight modification [35, 36]. The seeds were removed, and the fresh pulps of fruits were air-dried at room temperature (26 ± 1) in the shadow and then milled into a fine powder using an electric grinder. The powdered fruits were macerated in 500 ml ethanol and water (50% V/V) at room temperature (26 ± 1°C) for 48 hours with occasional shaking. Then, the extracts were filtered out through a filter paper (Whatman® No.4). The resulting liquid was concentrated at 40°C in a rotary evaporator and then kept in the incubator (40°C) to remove organic solvents resulting in the dry powder (the final yield of the procedure was about 13.2% w/w).

#### 2.2.2. Total Phenolic Content (TPC) of the Extract

Total phenolic content (TPC) of the extract was measured according to Folin–Ciocalteu (FC) method, which was described previously [37, 38] with minor modification. A fraction (100 *μ*l) of OD's ethanolic solution (20 *μ*g/mL) was mixed with equal water volume in a test tube. Next, about 200 *μ*L of FC reagent was augmented to the tube. Following the next 2 min, 2600 *μ*L of a 5% (w/v) sodium carbonate solution was added. The developed color was read at 760 nm using a MultiSpec UV-Vis spectrophotometer (Shimadzu, Tokyo, Japan). Estimation of phenolic compounds was carried out regarding the polyphenol reference calibration curve of the ethanolic solution of Gallic acid (GA) in a range of 0.5 to 10 mg/L [38–40]. The amount of TPC was expressed as mg of GA equivalent (GAE) per gram of dry extract. For blank, the same process was performed with 100 *μ*l of distilled water instead of extract.

### 2.3. *In Vivo* Study

#### 2.3.1. Animal Husbandry and Experimental Design

Animal experiments were conducted in the laboratory animal center of the University of Zabol, Zabol, Sistan, and Baluchistan Province, Iran. Twenty-four adult male Wistar rats (200 ± 20 g) were used in this study. Rats were housed in the condition of a temperature of about 26 ± 2 °C, controlled humidity, and a 12/12°h light/dark cycle. Rats had free access to taped water and a standard laboratory diet (Javaneh-Khorasan, Iran). The Animal Ethics Committee ethically approved the experimental procedures of the University of Zabol, Zabol, Sistan, and Baluchistan Province, Iran (ethical ID: IR.UOZ.REC.1398.1).

**Figure 2:**
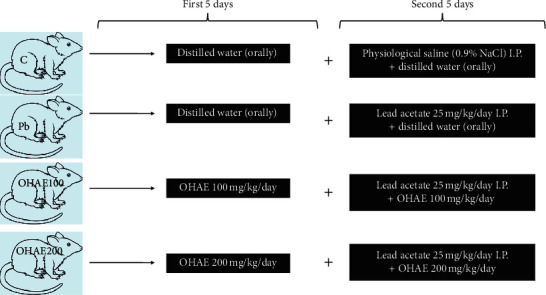
schematic diagram of *in vivo* study procedure.

The study protocol was conducted for ten days (first five days premedication + second five days premedication with Pb exposure, Figure 2). Animals were randomly and equally divided into four groups of six animals as follows:

1. Control group received daily distilled water orally (p.o.) for both the first and second 5 days but received physiological saline (0.9% w/v NaCl, i.p.) at the same volume of other groups daily during the second 5 days of the experiment
2. Pb group received daily administration of distilled water (p.o.) for both first and second 5 days and then received lead acetate (25 mg/kg b.w/day, i.p.) during the second 5 days of the experiment
3. OHAE100 received daily administration of *OHAE* (100 mg/kg b./day, p.o.) for both the first and second 5 days and then received lead acetate (25 mg/kg b.w/day, i.p.) during the second 5 days of the experiment
4. OHAE200 received daily administration of *OHAE* (200 mg/kg b./day, p.o.) for both the first and second 5 days and then received lead acetate (25 mg/kg b.w/day, i.p.) during the second 5 days of the experiment

Notably, groups 3 and 4 were also injected (i.p.) by 0.5 mL lead acetate at a dose of 25 mg/kg b.w./day for the second 5 days combined with *OD* fruit extract (Figure 2).

The dose of lead acetate was adjusted based on literature reports and preliminary studies [41]. Finally, rats fasted for 12 hours and then anesthetized with diethyl ether, and blood samples were collected by the retroorbital puncture using dry tubes. Blood samples were centrifuged (3000 rpm for 5 min) for separating the serum. The serum was immediately frozen at −80°C until use [40, 42].

#### 2.3.2. Serum Biochemical Parameters

Blood samples were collected using retroorbital puncture, and serum samples were collected as described in the animal husbandry and experimental design section. According to the manufacturer of the kit, the analyses of serum ALT, AST, and ALP levels were performed by relevant commercial kits using the Selectra pro, M autoanalyzer (Vital Scientific, SpanNeren, Netherlands).

#### 2.3.3. Evaluation of Lipid Peroxidation (TBARS and Catalase Activity)

MDA level (as TBARS) and catalase activity in serum samples were measured using the relevant commercial biochemistry kits (BioAssay Systems, USA and ZellBio Germany, respectively) according to the manufacturer's instructions.

#### 2.3.4. Histopathological Examination

After euthanasia, liver specimens were sliced and preserved in 10% formalin and processed for histological staining. After paraffin embedding and block making, serial sections were stained with hematoxylin-eosin and evaluated (Olympus, Tokyo, Japan) at 20, 40, and 100 magnifications. Liver sections were numerically graded from 0 to 4, covering no liver injuries to severe lead-induced hepatic injuries such as cytoplasmic vacuolation, cell necrosis, sinusoidal dilation, and hemorrhage based on the method previously described (Table. 1) [43]. Afterward, histopathological grading data was statistically tested using the Kruskal-Wallis test analysis followed by the *post hoc* Dunn's multiple comparisons.

### 2.4. *In Vitro* Study

#### 2.4.1. Cell Culture

The cells were cultured in DMEM/F12 plus 1% v/v of Pen/Strep (100×) and 10% v/v of heat-inactivated FBS supplemented with 0.5 *μ*g/mL amphotericin B and two mM L glutamine (all from Invitrogen, Carlsbad, CA, USA) under the condition of 37°C and 5% v/v CO_2_, in a humidified incubator.

#### 2.4.2. Cell Viability

The effect of various concentrations of the extract on cell viability was examined using MTT (3-(4,5-dimethyl-2-thiazolyl)-2,5-diphenyl-2H-tetrazolium bromide) proliferation assay color. Five thousand HepG2 cell lines were grown in a 96-well plate and treated with different concentrations of the extract at a range of 0–160 *μ*g/ml for 48 hours. Due to the hardy solubility of the extract at 160 *μ*g/ml, we used a lower concentration of the extract (80 *μ*g/ml) as the highest concentration for the study. Furthermore, another design was also done to evaluate the extract's protective impact on lead acetate-induced cell death. In review, the cells were pretreated at concentrations of 0–80 *μ*g/ml for 24 hours and then subjected to 100 *μ*g/ml of lead acetate [44] for a further 24 hours of coincubation. Afterward, 10 *μ*l of MTT reagent (5 mg/ml) was added to each well incubated for the next 3 hours. Formazan crystals were dissolved in 100 *μ*L DMSO, and the absorbance was read using StatFAX 2100 ELISA plate reader (Awareness Inc, USA) at 570 nm in referencing 620 nm. The assay was carried out six times and replicated three times for each sample [13, 45]. The final concentration of DMSO, as cosolvent, was lower than 0.1% v/v for all experiments.

#### 2.4.3. Evaluation of Total Glutathione (GSH), Lipid Peroxidation (MDA), and Inflammatory Cytokine (TNF-*α*)

GSH and MDA levels were measured using the relevant commercial biochemistry kits (ZellBio, Germany) according to the manufacturer's instructions [42]. As indexes of inflammation, TNF-a was also measured using the relevant ELISA kit (Bender Med, Germany). In brief, the cells were cultured at a density of 5,000,000 cells/mL overnight. Next, the cells were pretreated via different concentrations (20–80 *μ*g/ml) of the extract for 24 hours and subsequently were exposed to 100 *μ*g/ml of lead acetate for an additional 24 hours. Finally, the supernatants were separated for measuring each indicator using the manufacturer's instructions of the kit.

### 2.5. Statistical Analysis

Statistical analysis was performed using GraphPad Prism 8 for Windows (San Diego, CA). Data of oxidative stress and inflammation markers were expressed as mean ± standard deviation (SD) and analyzed by one-way analysis of variance (ANOVA) followed by Tukey's posttest. For the evaluation of histopathological scores, nonparametric analysis Kruskal-Wallis test was performed with the *post hoc* Dunn's multiple comparisons test and expressed as mean with range. Statistical significance was accepted at *p* ≤ 0.001, 0.01, and 0.05.

## 3. Results

### 3.1. Total Phenolic Content of the Extract

Generally, the FC method is often utilized for the evaluation of TPC in natural products. The value of TPC for the extract was 65 mg GAE/g dried extract.

### 3.2. Animal Study

#### 3.2.1. The Effects of OHAE on the Serum Levels of Liver Function Enzymes

**Figure 3:**
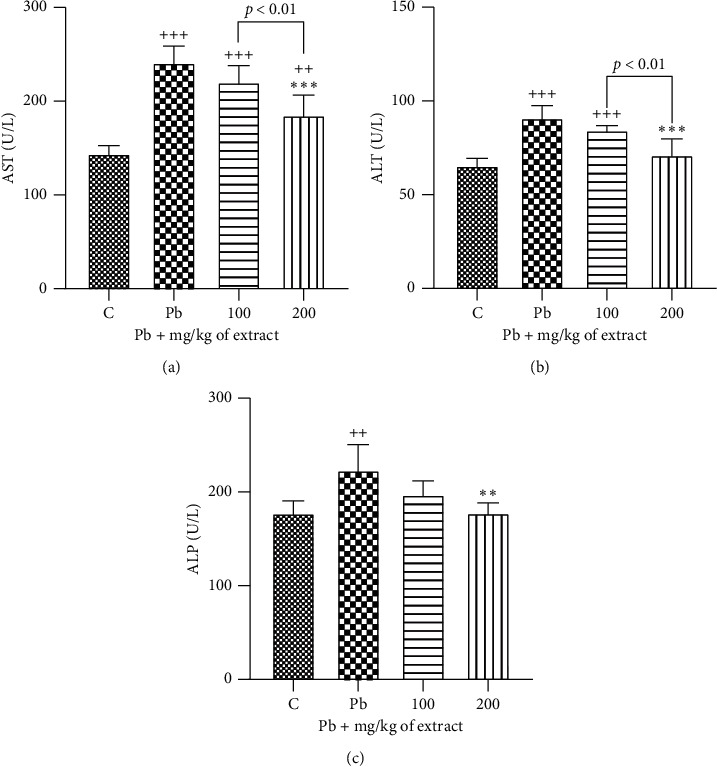
Effect of *OHAE* at 100 and 200 mg/kg on the activity levels of liver enzymes (a) AST, (b) ALT, and (c) ALP, following the lead toxicity. Values are expressed as mean ± SD. ^*∗∗*^*p* < 0.01, ^*∗∗∗*^*p* < 0.001 compared to Pb group; ^++^*p* < 0.01, ^+++^*p* < 0.001 compared to control group. The lines represent comparisons between extract treated groups.

The present data indicated that the treatment with lead acetate significantly increased serum AST, ALT, and ALP activities compared to the control (*p* < 0.001 to 0.01 for all cases). Meanwhile, treatment with both doses of *OHAE* (100 and 200 mg/kg b.w/day) markedly decreased these parameters compared to the lead acetate treated group (*p* < 0.001 to 0.01 for all cases) (Figures 3(a)–3(c)).

#### 3.2.2. The Effects of OHAE on the Serum Levels of TBARS and Catalase Activity

**Figure 4:**
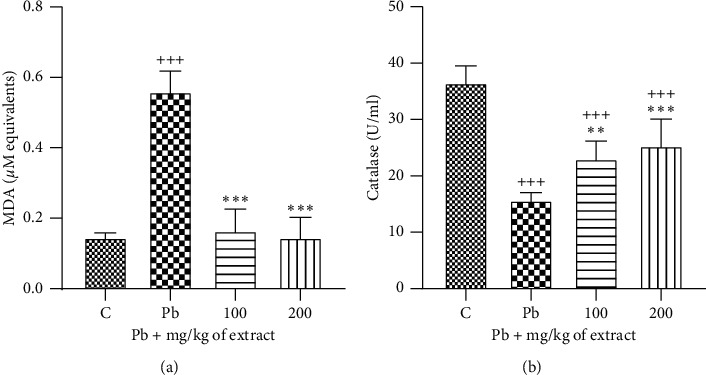
Effect of *OHAE* at 100 and 200 mg/kg on the levels of oxidant and antioxidant parameters: (a) MDA concentration and (b) catalase (CAT) activity, respectively, following lead toxicity. Values are expressed as mean ± SD. ^*∗∗*^*p* < 0.01 and ^*∗∗∗*^*p* < 0.001 compared to Pb group; ^+++^*p* < 0.001 compared to control group. The lines represent comparisons between extract treated groups.

Treatment with lead acetate significantly decreased serum catalase activity, while it increased serum MDA concentration compared to the control group (*p* < 0.001 for all cases, Figure 4(b)). Administration of *OHAE* (100 or 200 mg/kg b.w/day) before and during the lead acetate injection reversed these parameters compared with the Pb group (*p* < 0.001 to 0.01, Figures 4(a) and 4(b)).

#### 3.2.3. The Effects of OHAE on the Liver Histopathological Scores

**Figure 5:**
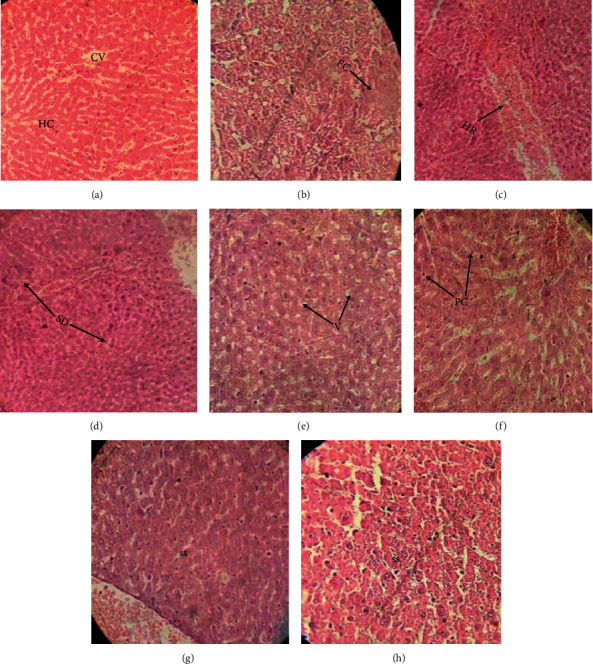
Paraffin sections stained by hematoxylin and eosin (H&E × 20 and ×40) for histopathological examination of liver tissues of rats as follows: normal hepatocytes architecture and sinusoids (HC) and central vein (CV) in the control group ((a) ×20). Alterations in liver tissue including fatty change (FC) ((b) ×40) and hemorrhage (HR) ((c) ×20) as well as the degenerated cells with sinusoidal dilatation (SD) ((d) ×20), cytoplasmic vacuolation (V) ((e) ×40), and pyknotic nuclei (PC) ((f) ×40) in Pb group. Reduction in lead-induced alterations resulted in regeneration of normal sinusoidal structures (SS) ((g) ×40) in the *OHAE*100 and ((h) ×40) in the *OHAE*200 groups.

The liver sections of lead acetate treated rats showed significant patterns of degenerated hepatic cords and fatty change (FC) (Figure 5(b)), hemorrhage (HR) (Figure 5(c)), sinusoidal dilatation (SD) (Figure 5(d)) as well as hepatic vacuolation (V) (Figure 5(e)), and pyknotic nuclei (PC) (Figure 5(f)) in comparison to the control group, in which normal hepatic cord and central vein architecture were obvious (CV and HC) (Figure 5(a)). Also, histological alterations were markedly reduced by the *OHAE* at 100 and 200 mg/kg/b.w/day (Figures 5(g) and 5(h), respectively).

**Figure 6:**
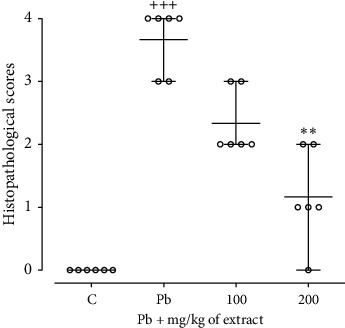
The histopathological score of liver injuries. Data are expressed as scatter dot plots and mean with range according to the nature of data (nonparametric). Accordingly, a nonparametric analysis Kruskal–Wallis test was performed with the *post hoc* Dunn's multiple comparisons test (*n* = 6 per group). ^+++^*p* < 0.001 compared to the control group. ^*∗∗*^*p* < 0.01 compared to the Pb group.

Overall, the pathological scores were notably more significant in the Pb group than in the control group (*p* < 0.001, Figure 6). In contrast, treatment with both doses of the extracts reduced the pathological scores compared to the Pb group, although these reductions were statistically significant only at 200 mg/kg of the extract compared to the Pb group (*p* < 0.01, Figure 6).

### 3.3. Cellular Study

#### 3.3.1. The Effects of OHAE on the Level of Cell Viability

**Figure 7:**
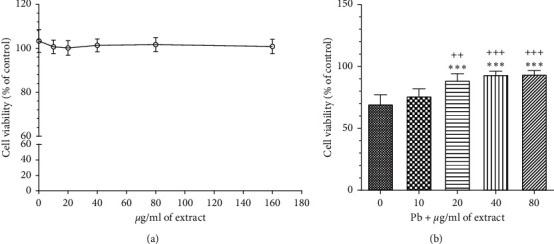
The effect of *OHAE* on HepG2 cell viability in the absence (a) and presence of lead acetate- (Pb-) induced hepatotoxicity (concentration: 100 *μ*g/ml, (b)). In Figure 7(a), five thousand HepG2 cell lines were grown in a 96-well plate and treated with different concentrations of the extract at a range of 0–160 *μ*g/ml for 48 hours. In Figure 7(b), the cells were pretreated at concentrations of 0–80 *μ*g/ml for 24 hours and then subjected to 100 *μ*g/ml of lead acetate for a further 24 hours of coincubation. ^*∗∗∗*^*p* < 0.001 compared to the group without extract. ^++^*p* < 0.01 and ^+++^*p* < 0.001 compared to the group incubated with 10 *μ*g/ml of extract.

Our findings demonstrated that different concentrations of the extract (0–160 *μ*g/ml) had no cytotoxicity on the HepG2 cell line (Figure 7(a)). Besides, pretreatment along with all tested concentrations of *OHAE* (20–80 *μ*g/ml) significantly increased cell viability against lead acetate toxicity (Pb, concentration: 100 *μ*g/ml) in a concentration-dependent manner in comparison to the Pb-treated group alone (*p* < 0.001, for all cases, Figure 7(b)).

#### 3.3.2. The Effects of OHAE on the Levels of Oxidative Stress Indices (GSH and MDA)

**Figure 8:**
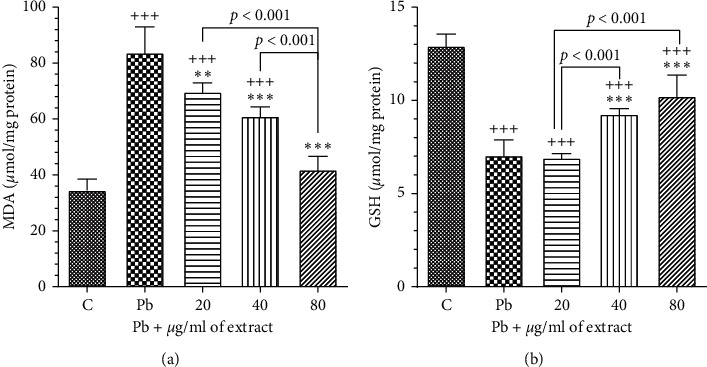
Effect of OHAE at 20, 40, and 80 *μ*g/ml on the levels of oxidant and antioxidant parameters (a) MDA and (b) GSH, respectively, following lead toxicity (100 *μ*g/ml). Values are expressed as mean ± SD. ^*∗∗∗*^*p* < 0.001 and ^*∗∗*^*p* < 0.01 significant compared to the Pb group; ^+++^*p* < 0.001 significant compared to the control group. The lines represent comparisons between extract-treated groups.

Incubation with lead acetate (100 *μ*g/ml) led to a significant increment in the level of MDA and significant decrement in the level of GSH compared to the control group (*p* < 0.001 for all cases, Figures 8(a) and 8(b)). However, *OHAE* treatment could significantly alleviate these values in all concentrations for MDA and 40 and 80 *μ*g/ml for GSH compared to the Pb group (*p* < 0.001 for all cases, Figures 8(a) and 8(b)).

#### 3.3.3. The Effects of OHAE on the Level of Inflammatory Cytokine (TNF-*α*)

**Figure 9:**
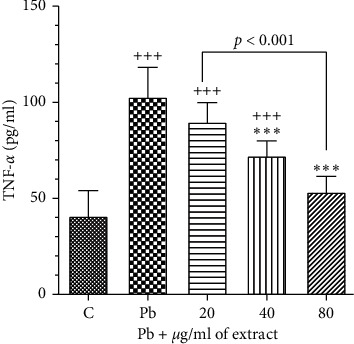
Effect of *OHAE* at 20, 40, and 80 *μ*g/ml on Pb (100 *μ*g/ml) induced inflammation. Values are expressed as mean ± SD. ^*∗∗∗*^*p* < 0.001 significant compared to the Pb group; ^+++^*p* < 0.001 significant compared to the control group. The lines represent comparisons between extract-treated groups.

Lead acetate (100 *μ*g/ml) caused a significant increase in the level of TNF-*α* (*p* < 0.001, Figure 9). However, *OHAE* treatment significantly reduced this inflammatory marker at 40 and 80 *μ*g/ml in comparison to the control group (*p* < 0.001 for all cases, Figure 9).

## 4. Discussion

To the best of our knowledge, this is the first study that examined the protective impacts of *OHAE* against lead acetate-induced liver toxicity by both animal and cellular evaluations. In this study, we evaluated lead acetate-induced hepatotoxicity and the protective impact of *OHAE* at 100 and 200 mg/kg through its antioxidant properties *in vivo* and antioxidant and anti-inflammatory characteristics *in vitro*. Briefly, our results showed that lead acetate caused liver injuries in both models, while the extract could retrieve the injuries.

Lead poisoning is known as a significant health problem in the world, especially in developing countries. Despite the enormous operational processes accomplished to control this burden, cases of lead toxicity still are observed [16]. Exposure to continuous and even low lead levels can exert liver, kidney, reproductive, and behavioral impairment, but it has been proved that the liver is the most susceptible organ to lead intoxication [46–49]. We observed that lead acetate significantly increased the serum levels of AST, ALP, and ALT compared to the control group, while the administration of *OHAE* significantly reduced these values. AST, ALT, and ALP are enzymes found highly concentrated in the liver, so the elevation of these serum parameters may be a precious indicator of liver injuries [50–53]. Elevation of these enzymes in serum may be partly due to the disruption of hepatocytes membrane integrity and leakage of these enzymes into the systemic circulation [54, 55].

Various mechanisms are defined for lead toxicity, but it seems that oxidative stress induced by lead accumulation is the most crucial reason for lead toxicity [16, 56]. Our *in vivo* study also showed that administration of lead acetate significantly developed oxidative stress mechanisms by the significant increment of MDA and, on the contrary, a significant decrement of serum catalase activity. In contrast, *OHAE* significantly reduced the serum content of MDA and increased catalase activity. An increase in MDA level as an indicator of lipid peroxidation and a decrease in catalase activity in serum are essential indicators of free radicals' production and provide an imbalance of oxidant/antioxidant system leading to lipid peroxidation in cell membranes of hepatocytes [57–61]. Therefore, impairment of cell membrane integrity of hepatocytes and leakage of transaminases and ALP in the serum in our study may be due to free radical interaction with the membrane's lipids, especially phospholipids. Previous studies have explained that lead toxicity is associated with liver damages and an elevation in the levels of transaminases and ALP and the histopathological parameters. Oxidative stress is another consequence of lead intoxication [62–68]. The result of these studies is concurrent with our study and can support our result regarding the models.

Our study indicated that *OD* extract (0–160 *μ*g/ml) had no cytotoxicity on the HepG2 cell line. Pretreatment of the lead-exposed cells with the extract (20–80 *μ*g/ml) could significantly retrieve cell viability in a concentration-dependent manner. The result of our study was in concordance with Jelena Katanić et al. that stated that different extracts of *OD* fruit (from 100 to 500 *μ*g/ml) had no cytotoxicity on the HepG2 cell line (IC_50_ > 500 *μ*g/ml) [69]. In another study, the *OD* cladodes also showed mild cytotoxic effects on murine macrophage cell line RAW 264.7 at the concentration of 100 *μ*M [70]. The fruits of *Opuntia robusta* and *Opuntia streptacantha*, two other plants of the *Opuntia* species, also showed significant protection against acetaminophen-induced cell death in the rat-isolated hepatocytes [71]. Bahira Harrabi and coauthors have also been reported that the polysaccharides obtained from the cladodes of *OD* (0–400 *μ*g/ml) did not possess any cytotoxicity. Meanwhile, these polysaccharides showed antioxidant activities through inhibition of 1,1-diphenyl-2-picrylhydrazyl (DPPH) and 2,2′-azino-bis(3-ethylbenzothiazoline-6-sulfonic acid (ABTS) radical-induced cytotoxicity at the concentration of 100 *μ*g/mL [72].

Additionally, lead is also known to reduce iron needed for the biosynthesis of heme, leading to the reduction of heme bioavailability and further the reduction of the activity of catalase [56, 73]. We revealed that *OHAE* could increase GSH level and catalase activity in both *in vitro* and *in vivo* experiments. Nataraj Loganayaki et al., in a study, tried to assess the antioxidant effects of different fruits [74]. They showed that *OD* fruit has a potent DPPH free radical scavenging activity, which describes plentiful sustainable hydrogen donating and radical scavenging ability (IC_50_ ≈ 43 *μ*g/ml) for *OD* fruit. They also stated that high levels of high molecular weight phenolics (tannins) observed in *OD* fruit are responsible for this effect [74]. Therefore, the *in vitro* antioxidant effect of *OHAE* in our study also is partly due to the high content of these high molecular weight phenolic compounds. We also evaluated the inflammatory status of the HepG2 cell line in the presence or absence of *OD* extract treatment against Pb-induced toxicity. The result showed that lead acetate provided a significant increment in the level of TNF-*α*, an essential indicator of inflammation [75]. Increased inflammation due to lead administration may be a critical consequence of increased oxidative stress through activation of NF-*κ*B [40].

*OD* fruits are composed of valuable chemical constituents with confirmed medicinal applications, including phenolics, betanidin, ascorbic acid, and minerals such as Na, Ca, Mg, Mn and Cr, K, Fe, Zn, and Ni [20]. Our study showed that *OHAE* treatments significantly alleviate lead acetate-induced injuries both *in vivo* and *in vitro* by reducing the levels of inflammatory and oxidative markers. Previous studies declared that *OD* fruit is an excellent source of betalains, especially betanidin and ascorbic acid, with desirable pharmacologic properties such as antioxidant, anti-inflammatory, anticancer, and antilipidemic effects [20, 76, 77]. Ascorbic acid (vitamin C) is known to contribute to carnitine biosynthesis, known as an anti-inflammatory agent [78, 79]. These data together highlight the anti-inflammatory and antioxidant effects of *OD* that preserve the cell membrane of hepatocytes from further damages [80, 81]. Luisa Tesoriere, in a clinical trial, stated that *Opuntia ficus-indica*, another fruit from the Opuntia family, is more potent than vitamin C in the reduction of oxidative stress that highlights our findings in the present study [82]. These OD fruits' properties were also studied in previous studies, which are in the same line with the result of our present study [20, 28, 77, 83, 84]. Phenolic compounds are other valuable compounds that are present in the *OD* fruits [20].

As limitations of the present study, lactate dehydrogenase (LDH) is an exciting marker to assess liver damage that we did not measure in the present study, suggesting further investigation. Additionally, the effects of lead exposure on hemoglobin heme group synthesis may reduce the functional capacity of cytochrome P-450 in the liver system. Thus, it would be suggested to evaluate the quality if other parameters are related to the production of hemoglobin and hematological changes would be evaluated in future work. In addition to liver damage, the effects of lead exposure on bones, the central and cardiovascular nervous systems, and the kidneys have been reported, which can be considered the extract's protective effects against them.

In conclusion, this study has demonstrated that *OHAE* significantly reduced lead acetate-induced liver injury, oxidative stress, and inflammation by the view of histopathology, *in vivo* and *in vitro*. These effects may be manifold by OD fruits' chemical composition, mainly phenolic, betacyanin, and mineral compounds found in OD fruits, which can be suggested for further consideration in other studies. Taken together, *OD* extract may have potential protective effects against lead toxicity as herbal medicine after clinical studies.

## Figures and Tables

**Table 1:** Pathological scoring approaches for liver injury based on the previously described by Abu-Amara et al. [43].

Score	Definition
0	Minimal or no evidence of injury
1	Mild injury, including cytoplasmic vacuolization and focal nuclear pyknosis
2	A moderate injury such as cytoplasmic vacuolization, areas of hepatocyte ballooning no necrosis, sinusoidal dilatation, and congestion, as well as the blurring of intercellular borders
3	Moderate to severe injury, areas of coagulative necrosis, cytoplasmic hypereosinophilia, severe sinusoidal dilatation, and congestion
4	Severe injury, including severe confluent coagulative necrosis and disintegration of and hemorrhage between hepatic chords, leads to loss of tissue architecture

## Data Availability

The data will be available by request to the corresponding authors.

## Abbreviations

ALP: Alkaline phosphatase
ALT: Alanine aminotransferase
AST: Aspartate aminotransferase
FBS: Fetal bovine serum
GA: Gallic acid
GSH: Glutathione
HepG2: Human liver cancer cell line
i.p.: Intraperitoneal
IHME: Institute for Health Metrics and Evaluation
IL-1*β*: Interleukin-1beta
IL-6: Interleukin-6
MDA: malondialdehyde
NF-*κ*B: Nuclear factor-kappa B
OD: *Opuntia dillenii* HAW
*OHAE*: *Opuntia dillenii* haw fruit hydroalcoholic extract
TBARS: Thiobarbituric acid reactive substances
TNF-*α*: Tumor necrosis factor-alpha
WHO: World Health Organization
DPPH: 1,1-diphenyl-2-picrylhydrazyl
ABTS: 2,2′-azino-bis(3-ethylbenzothiazoline-6-sulfonic acid).
